# Leprosy and Religion

**Published:** 1903-09-12

**Authors:** 


### LEPROSY AND RELIGION.

Mr. Jonathan Hutchinson brings forward a further series of figures in support of his thesis that the cause of leprosy is to be found in the consumption of imperfectly cured fish. The statements now submitted relate to the conditions existing in the United States of Colombia. The population of this South American Republic is, according to the new volumes of the "Encyclopædia Britannica," 4,000,000, and includes no fewer than 27,000 lepers, that is in round numbers 70 lepers in every 10,000 inhabitants, whereas in India, the so-called "land of the leper,"

the ratio is not more than 6 per 10,000. Mr. Hutchinson argues that this wide difference cannot possibly be explained by the theory of contagion. The Colombians do not expose themselves to risk in this direction more than the Hindus, and during the last 30 years far more energetic measures of segregation have been adopted in Colombia than in India. On the other hand, the practice of fish-eating occupies a very different position in the two countries. In India large sections of the population have a prejudice against fish as food, whilst in Colombia the consumption of fish receives an artificial stimulus from the ritual of the Roman Catholic Church. Further, the inland districts of Colombia are in many instances very deficient in a natural supply of fish, and this, Mr. Hutchinson urges, means, under the influence of an artificial demand, the creation of a supply of fish food in a bad condition. Whatever may be the truth in this question it is noteworthy that Mr. Hutchinson is able to produce large groups of facts from various parts of the world in support of his views, and that whilst the leading authorities reject his theory no prominent champion comes forward to explain away the facts on which his doctrine is founded.

